# Effects of the Application of *Lactobacillus plantarum* Inoculant and Potassium Sorbate on the Fermentation Quality, In Vitro Digestibility and Aerobic Stability of Total Mixed Ration Silage Based on Alfalfa Silage

**DOI:** 10.3390/ani10122229

**Published:** 2020-11-27

**Authors:** Yixiao Xie, Shengyang Xu, Wenqi Li, Musen Wang, Zhe Wu, Jinze Bao, Tingting Jia, Zhu Yu

**Affiliations:** 1 College of Grassland Science and Technology, China Agricultural University, Beijing 100193, China; bs20183040396@cau.edu.cn (Y.X.); sy20193040640@cau.edu.cn (S.X.); lwqnd@cau.edu.cn (W.L.); wuzhe@cau.edu.cn (Z.W.); SY20183040590@cau.edu.cn (J.B.); B20163040303@cau.edu.cn (T.J.); 2 State Key Laboratory of Grassland and Agro-Ecosystems, School of Life Sciences, Lanzhou 730000, China; wangms@lzu.edu.cn

**Keywords:** TMR silage, potassium sorbate, *Lactobacillus plantarum*, fermentation quality, in vitro digestibility, aerobic stability

## Abstract

**Simple Summary:**

Ensiling total mixed ration allows preservation and saves labor for small farms. This study evaluated the substitution relationship between lactic acid bacteria (*Lactobacillus plantarum*) and silage components, and verified the practicality of preservative (potassium sorbate) in total mixed ration silage. The results showed that potassium sorbate greatly improved the preservation efficiency of total mixed ration silages. The alfalfa silage could directly produce an acidic environment for fresh total mixed ration before ensiling and showed comparable function to inoculant in the improvement of fermentation quality. Therefore, the application of the inoculant is not necessary when the total mixed ration contains a certain percentage of silage. These findings could provide guidance for farmers to avoid the blind use of inoculants and the spoilage of total mixed ration silage, which could directly improve economic efficiency.

**Abstract:**

This study aimed to evaluate the effect of the application of an inoculant and a preservative on the fermentation quality, in vitro digestibility, and aerobic stability of alfalfa silage-based fermented total mixed ration (TMR). The TMR was ensiled with (1) no additives (control), (2) *Lactobacillus plantarum* (LP), or (3) potassium sorbate (PS). The V-scores of all silages were higher than 80 points during the 30 days of ensiling. The addition of LP and PS had no effects on the in vitro parameters, such as in vitro digestibility and in vitro gas production (*p* > 0.05). LP-treated silage showed similar fermentation quality and comparable aerobic stability to the control (110 h). The LP only decreased the ammonia nitrogen (NH_3_-N) content (*p* < 0.05) during ensiling. The PS significantly increased the pH of TMR silages (*p* < 0.05). Meanwhile, the addition of PS improved the aerobic stability (>162 h) of TMR silage, indicated by the higher water-soluble carbohydrate content and lower NH_3_-N content in comparison with those in the control after aerobic exposure (*p* < 0.05). The improvement in fermentation quality is extremely small in terms of applying LP in TMR silage based on a large percentage of other silage ingredients. The PS is effective in conserving unpacked TMR silage and showed the potential to reduce the risk of ruminal acidosis in livestock.

## 1. Introduction

Silage is a product based on fermentation, whereby lactic acid bacteria (LAB) convert water-soluble carbohydrates (WSC) to organic acids under anaerobic conditions. Additives such as LAB, chemicals, and enzymes are often applied in silage to enhance its preservation [1]. Well-preserved silage has dominant lactic acid content, low NH_3_-N content, and negligible butyric acid content [2]. The appropriate adjustment in density, moisture content, chopping length, and the application of additives can significantly improve the fermentation quality, digestibility, and aerobic stability of silage.

Total mixed ration (TMR) is a form of complete formula feed consisting of roughage, concentrate, minerals, vitamins, and other additives in certain proportions. It is widely used to provide ruminants with adequate and balanced nutrition, which can stabilize microbial function and enhance energy and protein utilization in the rumen [3]. However, the processing of TMR requires professional equipment or sufficient labor. Fresh TMR is also a highly deteriorative feedstuff that cannot be preserved for long periods. Ensiling can prevent the spoilage of TMR and improve its palatability by anaerobic fermentation [4]. Baled TMR silages can be transported to provide year-round nutritional balance feed for small-scale farms that lack labor.

Silage is also a common roughage in TMR, which has low pH and a large number of lactic acid bacteria attached to it. Fermented feedstuffs have been successfully used as raw materials for TMR silage in industry [5]. Sometimes a part of protein feed may be replaced by relatively inexpensive alfalfa (*Medicago sativa*) silage with poor quality in TMR production to reduce feeding costs. Alfalfa silage is favorable for ruminal fermentation and can also improve the dry matter intake of cows [6]. Nishino et al. [7] found that the LAB species in TMR silages were selected during the ensiling process, and the bacterial community was unrelated to the ingredient crop silages. It was not conclusive as to whether the silage could directly stabilize the fermentation of TMR silage when the single silage composition accounted for more than half of the dry matter of the ingredients. *Lactobacillus plantarum* (LP) has been added to TMR silage and has proven to be effective in altering fermentation characteristics [8,9]. However, few studies have investigated whether it is necessary to reinoculate LP for TMR silage based on a large percentage of other silage ingredients, as well-fermented silage might already play the role of inoculant. Furthermore, the main factor limiting the use of TMR silage by ruminants is the digestibility of the fiber. In an early study, LP improved the in vitro dry matter digestibility (DMD) of TMR silages [9]. Thus, an inoculant of LP was applied in alfalfa silage-based TMR silage to estimate the improvement of digestibility and to verify the necessity of inoculation.

TMR silages are inherently unstable after re-exposure to air during the feed-out phase [10]. It is a common scenario that unpacked TMR silage bales cannot be completely fed in time. It may take more than 5 days to consume an 800–1000 kg TMR silage bale for some small-scale family farms. Potassium sorbate (PS) is a typical inexpensive preservative. The efficiency of PS in silage preservation has also been demonstrated for a variety of crops, such as fescue, *Leymus chinensis*, and corn [11,12,13]. Therefore, PS can be a potential antiseptic factor for the TMR silage bale. However, to the best of our knowledge, no research has investigated the effect of PS on TMR silage preservation. This study aimed to estimate the performance of PS in the preservation of TMR silages.

Therefore, this article reports on the effects of applying LP inoculant and PS on the fermentation quality, in vitro digestibility, and aerobic stability of TMR silage based on alfalfa silage.

## 2. Materials and Methods

### 2.1. Total Mixed Ration (TMR) Silage Preparation

The chemical composition of the ingredients and the composition of the TMR are given in Table 1 and Table 2, respectively. First-cutting alfalfa (*Medicago sativa* L) was harvested at the full-bloom stage in Guyuan, Ningxia, China (106°17′ E, 36°28′ N, elevation 1529 m), on 19 June 2019. The wilted alfalfa was chopped to a length of 2–5 cm and baled without any additives by a round baler (Comprima, Krone, Germany). Alfalfa silage bales were unpacked after 55 days of ensiling. Crushed corn cob and corn grain were obtained from a private medium-scale cattle farm in Guyuan, Ningxia, China. The mixed concentrate was purchased from Botai Company (Guyuan, China) and was composed of soybean meal, rapeseed meal, cotton meal, corn gluten meal, distillers’ dried grains, corn peel, and vitamin–mineral mix. TMR mixtures (750 g, fresh matter) were mixed well and then tightly packed into plastic silos (1 L). The TMR was treated with (1) distilled water (control), (2) LP, and (3) PS. The application rate of LP and the concentration of PS applied to each TMR were 10^6^ colony-forming units (cfu) g^−1^ and 2 g kg^−1^ on a fresh matter (FM) basis, respectively. Additives were dissolved in water, and the control treatment was sprayed with an equal amount of water. There were 15 silos for each treatment, among which triplicate silos were opened on days 7 and 14 for TMR silage fermentation quality determination, while the other 9 silos were opened on day 30 for both TMR silage quality determination and the aerobic stability test. Anaerobic fermentation was conducted at an ambient temperature of 22–28 °C. At the time of ensiling, triplicate silos of well-mixed TMR were taken as 0-day TMR silages for initial characterization analysis.

### 2.2. Chemical and Microbiological Analyses

The contents of each silo were removed and blended thoroughly after opening. The dry matter (DM) content was determined by a forced-draft oven at 65 °C for 48 h. The dried samples were ground to allow them to pass through a 1.0-mm screen and were used for chemical analysis. The crude protein (CP) content was determined according to the Kjeldahl procedure [14] and calculated as total N × 6.25. Examination of the ether extract (EE) was conducted by the method of the Association of Official Analytical Chemists (AOAC) [15]. The WSC content was analyzed by the anthrone–sulfuric acid method [16]. Neutral detergent fiber (aNDF) and acid detergent fiber (ADF) analyses were performed following the procedure described by Van Soest et al. [17]. Sodium sulfite and α-amylase were applied for aNDF determination, and both the aNDF and ADF content reported include residual ash.

TMR silage samples (20 g) from each silo were mixed with 180 mL of sterilized distilled water and then homogenized for 2 min in a blender jar. The mixtures were then filtered through 4 layers of cheesecloth and filter paper. The filtrate was centrifuged at 10,000× *g* at 4 °C for 10 min to analyze the pH and ammonia nitrogen (NH_3_-N) content. The pH was measured using an electrode (PHS-3C, INESA Scientific Instrument, Shanghai, China). The NH_3_-N content was determined according to the sodium hypochlorite and phenol method [18]. The supernatant was further processed with a 0.22-μm dialyzer to analyze organic acids. Lactic, acetic, propionic, and butyric acids were analyzed by high-performance liquid chromatography according to Tian et al. [19]. The V-score was calculated to evaluate the silage quality according to the volatile fatty acid and NH_3_-N contents [20]. Enumerations of LAB, yeasts, molds, and coliform bacteria were performed from fresh TMR silages using the method of Wang et al. [21].

### 2.3. In Vitro Incubation and Degradability Measurement

In vitro fermentation was carried out in Ankom RFS bottles using the pressure transducer technique (Ankom Technologies, Macedon, NY, USA) as described by Yuan et al. [4]. Rumen fluid was collected from 4 dry Holstein cows and kept at 39 °C. These cows were fed 3.5 kg (FM) of wheat straw, 2.8 kg of oat hay, 11 kg of whole-plant corn silage, 1 kg of rapeseed meal, 1 kg of soybean meal, 0.5 kg of distiller’s dried grains with solubles, 1.3 kg of corn peel, 0.05 kg of urea, and 0.2 kg of 5% premix. Filtered rumen fluid was mixed with buffer at a ratio of 1:4. The buffer consisted of 1330 mL of buffer A (KH_2_PO_4_, 10.0 g/L; MgSO_4_·7H_2_O, 0.5 g/L; NaCl, 0.5 g/L; CaCl_2_·2H_2_O, 0.1 g/L; and urea, 0.5 g/L) and 266 mL of buffer B (Na_2_CO_3_, 15.0 g/L and Na_2_S·9H_2_O, 1.0 g/L). Approximately 1 g of ground samples from 30-day TMR silage were added to rumen fluid–buffer mixtures in Ankom RFS bottles under CO_2_. The mixtures were incubated at 39 °C, and gas production in the bottles was measured every hour for 48 h. The blank was 3 RFS bottles with the only inoculum added. Cumulative gas production data were fitted to a gas production model modified from the Gompertz growth equation [22]:

V(*t*) = V(∞) exp [−exp (*ke* (λ − *t*)/V(∞) + 1)] (1)

where V*(t)* is cumulative gas production (mL), V(∞) is maximal cumulative gas production (mL), *k* is maximum gas production rate (mL·h^−1^), λ is lag time (h), *t* is time elapsed (h), and *e* is exponential of 1 (2.718).

In vitro DMD and in vitro neutral detergent fiber digestibility (NDFD) were determined with an Ankom Daisy^II^ incubator (Ankom Technologies, Macedon, NY, USA). Approximately 0.5 g of ground samples was put into the same fluid–buffer mixtures and incubated under CO_2_ for 48 h. The reduced weight of TMR samples was used to calculate the in vitro DMD. The aNDF content of the residue after incubation was also determined to calculate the in vitro NDFD.

### 2.4. Aerobic Stability Test

After 30 days of ensiling, 9 silos of each treatment were opened, and every third silo was mixed thoroughly and taken as the 30-day TMR silage sample (also as the 0-day aerobic exposure sample) for fermentation quality analysis. The remaining TMR silage of the 3 silos was placed into 4 new 1 L plastic silos without compaction (marked with days 1, 3, 5, and 7). The new silos were covered with 4 layers of cheesecloth and stored at ambient temperature (25 °C). A total of 12 silos marked with different exposure times for each treatment were exposed to air for the aerobic stability test.

A multichannel data logger (MDL-1048A, Tianhe, Shanghai, China) was used to record the temperature of the exposure samples every half hour. Aerobic stability was defined as the time before TMR silage temperatures increased by 2 °C above ambient temperature. Triplicate silos of aerobic exposure samples were collected as marked after 1, 3, 5, and 7 days to determine the pH and NH_3_-N, organic acid, DM, CP, and WSC levels, as well as microbial counts.

### 2.5. Statistical Analyses

Microbial data were log_10_-transformed and presented on a wet-weight basis. The data were subjected to one-way or two-way analysis of variance with fixed effects of ensiling time (or exposure time) and additives, analyzed by SPSS version 19.0 for Windows (SPSS Inc., Chicago, IL, USA). Duncan’s multiple range method was used to judge the differences among the means of the treatments and ensiling days (or exposure days). Means were considered significantly different at *p* < 0.05.

## 3. Results

### 3.1. Fermentation Quality of TMR Silages

The changes in fermentative characteristics of TMR silages during ensiling are presented in Table 3. The interaction between additives and ensiling period significantly affected (*p* < 0.05) the pH; lactic acid, acetic acid, propionic acid, and NH_3_-N levels; and V-score. The effect of additives on the pH; lactic acid, propionic acid, and NH_3_-N levels; and V-score of the TMR silages during ensiling was evident (*p* < 0.05). The days of ensiling also had a significant effect (*p* < 0.05) on all fermentative characteristics except butyric acid.

During the ensiling procedure (30 days), the organic acid contents in all silages gradually increased (*p* < 0.05). LP silages showed lower NH_3_-N content than other silages (*p* < 0.05). The NH_3_-N content decreased during the first 7 days, but increased during the next 7 days of ensiling (*p* < 0.05). The pH of the PS silages remained at 4.72 in the first 14 days, while the pH of the control and LP silages were both lower than 4.32 after 7 days of ensiling. The content of acetic acid in all silages increased greatly during days 14–30 (*p* < 0.05). The PS silages showed significantly higher (*p* < 0.05) WSC content than the other silages (Table 4). No butyric acid was detected in TMR silages during the 30 days of ensiling.

### 3.2. In Vitro Degradability of TMR Silages

**Figure 1:**
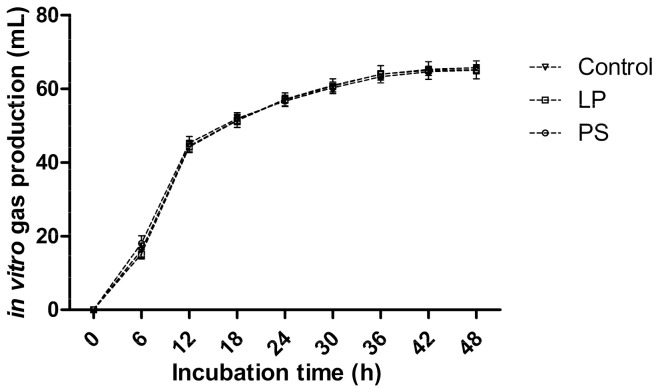
Gas production profiles from in vitro fermentation of the TMR silage for 48 h (bars indicate standard errors of the means). LP, *Lactobacillus plantarum* inoculant; PS, potassium sorbate.

The in vitro gas production profiles and in vitro parameters of 30-day TMR silages are presented in Figure 1 and Table 4. The curves for gas production of different treatments almost coincided. The additives showed no effects on in vitro DMD, in vitro NDFD, in vitro gas productions, and in vitro maximum gas production rate (*p* > 0.05).

### 3.3. Aerobic Stability of TMR Silages

The changes in the fermentative characteristics and chemical compositions of TMR silages during the aerobic exposure period are presented in Table 5. Significant interactions between additives and days of aerobic exposure were observed for the pH, CP, and NH_3_-N levels and V-score (*p* < 0.05). The additives significantly affected the pH; CP, WSC, lactic acid, and NH_3_-N levels; and V-score (*p* < 0.05). The days of aerobic exposure had a significant effect on the CP and WSC content and fermentative characteristics (*p* < 0.05), except for propionic acid and butyric acid. 

The pH of the control and LP silages significantly increased (*p* < 0.05) after 5 days of aerobic exposure, and it even increased above 7.50 with a substantial decrease in lactic acid content after 7 days of aerobic exposure. At the same time, the CP levels of the control and LP silages also decreased with a sharp increase in NH_3_-N content, and the V-scores of the control and LP silages were much lower than the PS silages (*p* < 0.05). Aerobic exposure had a relatively weak effect on the chemical compositions or fermentative characteristics of PS silages. TMR silages treated with PS also preserved greater amounts of WSC than other silages from treatments (*p* < 0.05). Additives showed no significant effect on DM content (*p* > 0.05). The levels of acetic acid and propionic acid in all silages steadily decreased during aerobic deterioration. No butyric acid was detected in TMR silages during the 7 days of air exposure.

**Figure 2:**
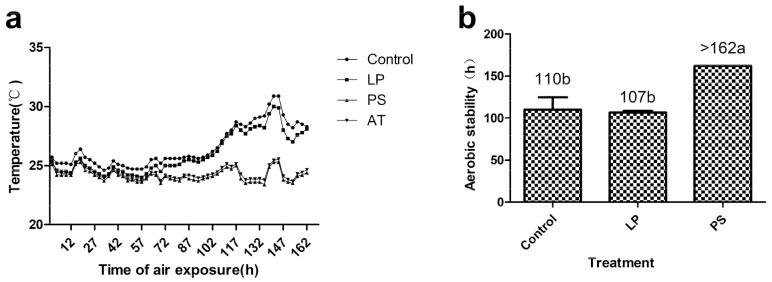
Dynamic changes in temperatures (**a**) and hours of aerobic stability (**b**) of TMR silages during air exposure (bars indicate standard error of the means). Values with different letters show significant differences among the treatments (*p* < 0.05). LP, *Lactobacillus plantarum* inoculant; PS, potassium sorbate; AT, ambient temperature.

**Figure 3:**
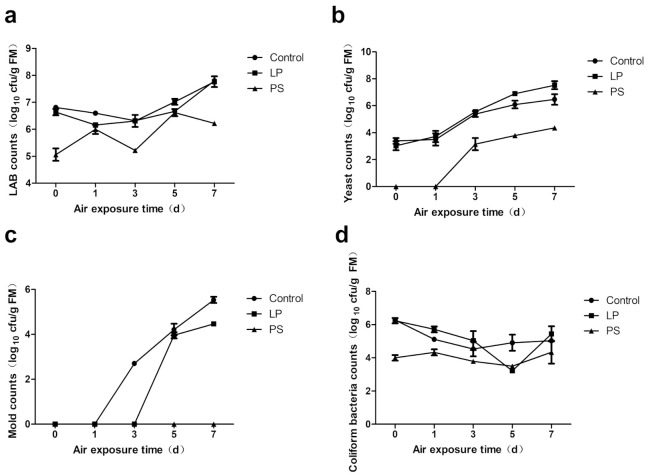
Dynamic changes in lactic acid bacteria (LAB) counts (**a**), yeast counts (**b**), mold counts (**c**), and coliform bacteria counts (**d**) of TMR silages during the processing of aerobic deterioration (bars indicate standard error of the means). cfu, colony-forming units; FM, fresh matter; LP, *Lactobacillus plantarum* inoculant; PS, potassium sorbate.

The dynamic changes in temperature during aerobic exposure are presented in Figure 2a. The periods during which TMR silages remained aerobically stable ranged from 107 to >162 h (Figure 2b). LP silages showed almost the same temperature trend and aerobic stability duration as control silages. The temperature of PS silages did not increase in the present experiment. Dynamic changes in viable microbial counts during aerobic exposure are shown in Figure 3. All the microbial counts of PS silages were lower than those of other groups (*p* < 0.05). The molds in the control silage were detected after 3 days, while those in the LP silage were detected after 5 days.

## 4. Discussion

### 4.1. Effects of Additives on the Fermentation Quality of TMR Silages

All TMR silages were well preserved during fermentation. Due to the low NH_3_-N content and lack of butyric acid detected in all silage samples, the V-scores of all silages were higher than 80 points, indicating good silage quality [2]. The significant interaction between the additives and ensiling days for V-score, organic acids, and NH_3_-N content might have been due to the different fermentation levels of the TMR silage at the seventh day. Due to the reductions in acetic acid and propionic acid content, all silages exhibited the highest V-score after 7 days of ensiling.

Generally, ensiling is performed to inhibit the growth of detrimental anaerobes by decreasing the pH, and inoculants such as LP could be used to accelerate this procedure. However, TMR silages could cause ruminal acidosis if the pH is too low, especially when the TMR silage contains high grain content, which is a cause for concern. Some buffer salts, such as ammonium formate, propionate, and sodium bicarbonate, were applied in silages in early studies, but none of these salts increased the pH of silage [23,24]. However, the PS showed an inhibitory effect on the growth of LAB in this study. The addition of PS decreased the contents of lactic acid and increased the pH of TMR silage during ensiling, which may reduce the risk of ruminal acidosis in livestock. The weaker fermentation in PS group preserved more WSC and also explained the significant interaction between the additives and ensiling days for pH. Ethanol has been proven to have a similar effect on the pH of TMR silage, but the cost may be higher than that of PS due to the addition of 25 mL kg^−1^ [4]. Further animal studies are required to judge the specific influence of PS-treated TMR silage in the rumen environment.

The NH_3_-N content of LP silages was lower than that of the control, suggesting that the inoculant outgrew the epiphytic LAB from alfalfa silage and dominated the fermentation, which reduced ammonia production from proteolysis [10,25]. All NH_3_-N levels decreased after 7 days of fermentation. These results may be due to some aerobic microorganisms from alfalfa silage or other ingredients that degrade NH_3_-N remaining active or becoming active again, such as *Bacillus* sp. strains [26]. Before the residual oxygen was completely consumed, the aerobic microorganisms and lactic acid bacteria may have degraded or utilized a portion of the remaining NH_3_-N, which was mainly derived from the unpacked alfalfa silage. The NH_3_-N content increased again after the aerobic microorganisms were inhibited. A similar result was reported by Anjos et al. [27], who found relatively low NH_3_-N content in re-ensiled sorghum silage. This finding may provide a strategy to reduce NH_3_-N content in silage-based TMR silages. However, the pH of the 0-day TMR silage was low, and no significant difference was found in the organic acid or WSC content between LP silages and control silages after 30 days of ensiling. The inoculation rates of the LAB strains are usually 10^5^–10^6^ cfu g^−1^ on an FM basis, which is often sufficient for the inoculants to become the predominant population in the TMR silage [25]. In contrast, the LAB counts in well-fermented alfalfa silage were proven to be much higher than 10^6^ cfu g^−1^ on an FM basis [28,29]. *Lactobacillus,* which plays a critical role in fermentation, usually dominates the bacterial community in alfalfa silage [30]. The results suggested that the alfalfa silage could directly produce an acidic environment for fresh TMR before ensiling and had a comparable function to LAB inoculant in the improvement or stabilization of fermentation, except for NH_3_-N content. In future experiments, we will investigate the specific percentage of silage that can play the role of inoculant in the TMR silage.

### 4.2. Effects of Additives on the In Vitro Degradability of TMR Silages

Digestibility is commonly accepted as a measure of feed nutritional value and intake [2]. Some studies showed that LP has a promoting effect on the digestibility of TMR silages [8,31,32], whereas other studies showed that LP did not have such an effect [4,33,34]. The contradictions and inconsistencies may be caused by the other different characteristics of the LP inoculants or the different composition of TMR silage. Neither PS nor LP improved the in vitro digestibility of TMR silages after ensiling in this study. Filya et al. [35] found that DMD was correlated with various fiber constituents. Both aNDF and ADF contents of the treated groups were similar to those of the control group. Similar results were reported by Zhao et al. [2]. In vitro gas production can be used to estimate the rate and extent of ruminal DM degradation [36]. Meanwhile, this parameter is also an indicator that can be used for the prediction of DM intake [37]. However, there was no significant difference in any of these in vitro parameters, which could also be due to the similar fiber constituents among the ensiled TMR silages. In fact, gas production and DMD are also positively correlated [38]. The results indicated that the addition of PS or LP had no adverse or beneficial effects on rumen utilization of the TMR silage.

### 4.3. Effects of Additives on the Aerobic Stability of TMR Silages

Many experiments have proven that TMR silage with sufficient fermentation will have higher aerobic stability than fresh TMR [33,39,40]. In the present study, the aerobic stability of untreated TMR silage was less than 5 days, which may have been due to the relatively simple composition of the ingredients [40]. The short aerobic stabilization time further proved the necessity of applying preservatives in some TMR silages. PS is a well-known additive in the conservation of a variety of feeds because of its antifungal properties [12]. Yeast is the key factor in the aerobic deterioration of silage, and the silage more easily deteriorated when the number of yeasts reached 10^5^ cfu g^−1^ (FM) [41]. Many yeast species could oxidatively metabolize lactic acid via the tricarboxylic acid cycle, which decreased the acidity of TMR silage and allowed for the growth of spoilage bacteria [42]. The PS inhibited the growth of yeasts and stabilized the acidic environment of silage. Therefore, TMR silages with PS showed low counts (<10^5^ cfu g^−1^ FM) in all detrimental microorganisms and high aerobic stability during this aerobic stability test. Meanwhile, PS preserved more WSC and lactic acid, reduced silage protein breakdown, and kept the NH_3_-N content stable during the whole process of aerobic exposure, which directly contributed to high V-scores until the end of the test. Therefore, the significant interaction between additives and aerobic exposure day for pH, V-score, NH_3_-N, and CP content was due to the inhibition of yeasts in PS silage. Specifically, the PS silage showed better fermentation quality at the seventh day of exposure.

Numerous studies have demonstrated that inoculation with homofermentative LAB reduces the aerobic stability of silages [8,11,43]. However, the LP group showed aerobic stability similar to that of the control group in this study. Lactic acid and WSC were potential sources of available substrate for the growth of undesirable microorganisms, and their levels were negatively correlated with aerobic stability [44]. The comparable lactic acid, WSC, and protective volatile fatty acid contents of LP and control silages before exposure may partly account for the similar aerobic stability. The LP strain did not inhibit the growth of yeast, which was the main cause of aerobic spoilage [44], and this result is in agreement with the results of Cai et al. [45]. In previous research by Filya et al. [43], both corn and sorghum silages inoculated with LP showed higher mold counts than control silages after 5 days of aerobic exposure. However, it is hard to explain why the addition of LP delayed and inhibited the growth of mold in comparison with the control in this study. We interpret that the LP silages produced some substances with antioxidant and antifungal properties during ensiling. Out of curiosity, we tested the phenolic acid contents of the 30-day TMR silages. We found that 11.0% and 18.8% more ferulic acid was extracted from the LP silage than from the control and PS silages, respectively, which may partially explain the inhibition of mold growth of LP silage. Additional research is needed for further verification of the antifungal properties of the silages treated with LP.

## 5. Conclusions

The addition of LP and PS did not improve the digestibility of TMR silage in this study. Although LP slightly decreased the NH_3_-N content and inhibited the growth of molds during aerobic exposure, the alfalfa silage showed comparable function to LAB inoculant in the improvement of fermentation quality. When TMR silage contains enough single silage ingredient to stabilize fermentation, there is no need to apply LP. The PS greatly improved the aerobic stability of TMR silages and showed the potential to reduce the risk of ruminal acidosis in livestock by increasing the pH of the silages.

## Figures and Tables

**Table 1:** Chemical composition and fermentation characteristics of ingredients used for the total mixed ration.

Item ^1^	Alfalfa Silage	Corn Cob	Corn Grain	Mixed ^2^ Concentrate
Dry matter (g kg^−1^ FM)	446.82	940.04	887.88	921.03
Crude protein (g kg^−1^ DM)	145.83	35.87	83.01	325.29
Neutral detergent fiber (g kg^−1^ DM)	423.30	810.26	98.50	263.31
Acid detergent fiber (g kg^−1^ DM)	296.02	417.88	38.09	110.84
Ether extract (g kg^−1^ DM)	41.69	38.21	48.84	58.09
Lactic acid (g kg^−1^ DM)	42.47	-	-	-
Acetic acid (g kg^−1^ DM)	4.56	-	-	-
Propionic acid (g kg^−1^ DM)	10.00	-	-	-
Butyric acid (g kg^−1^ DM)	ND	-	-	-
Ammonia nitrogen (g kg^−1^ TN)	31.06	-	-	-

ND, not detected. ^1^ FM, fresh matter; DM, dry matter; TN, total nitrogen. ^2^ The mixed concentrate was purchased from Botai Company (Guyuan, China) and was composed of soybean meal, rapeseed meal, cotton meal, corn gluten meal, distillers’ dried grains, corn peel, and vitamin–mineral mix.

**Table 2:** Ingredient composition and chemical composition of the total mixed ration.

Item ^1^	TMR
Ingredient composition (g kg^−1^ DM)	
Alfalfa silage	600
Corn cob	60
Corn grain	240
Mixed concentrate ^2^	100
Total	1000
Chemical composition (g kg^−1^ FM)	
Dry matter	487.51
Crude protein	146.18
Water soluble carbohydrate	15.33
Neutral detergent fiber	349.61
Acid detergent fiber	231.16

^1^ DM, dry matter; FM, fresh matter. ^2^ The mixed concentrate was purchased from Botai Company (Guyuan, China) and was composed of soybean meal, rapeseed meal, cotton meal, corn gluten meal, distillers’ dried grains, corn peel, and vitamin–mineral mix.

**Table 3:** Changes in fermentative characteristics during ensiling of total mixed ration (TMR) silages.

Item ^1^	Treatment ^2^	Days of Ensiling				SEM	*p*-Value ^3^		
		0	7	14	30		D	T	D × T
Fermentative Characteristics									
pH	Control	4.65 ^A^	4.32 ^bB^	4.3b ^B^	4.26 ^bB^	0.01	<0.001	<0.001	<0.001
	LP	4.70 ^A^	4.26 ^bB^	4.24 ^bB^	4.25 ^bB^				
	PS	4.67 ^A^	4.70 ^aA^	4.72 ^aA^	4.59 ^aB^				
LA	Control	36.12 ^D^	53.27 ^aC^	64.43 ^aB^	75.44 ^aA^	0.77	<0.001	<0.001	<0.001
	LP	30.05 ^C^	39.47 ^bB^	71.91 ^aA^	69.39 ^aA^				
	PS	33.47 ^C^	18.07 ^cD^	51.05 ^bB^	56.23 ^bA^				
AA	Control	3.56 ^B^	3.16 ^aB^	4.09 ^bB^	12.06 ^A^	0.10	<0.001	0.230	0.009
	LP	3.27 ^C^	2.39 ^aC^	5.15 ^aB^	12.25 ^A^				
	PS	3.44 ^B^	1.09 ^bC^	4.61 ^abB^	12.39 ^A^				
PA	Control	9.83 ^AB^	8.31 ^aB^	9.89 ^bAB^	12.51 ^A^	0.20	<0.001	0.008	<0.001
	LP	9.77 ^B^	5.84 ^abC^	5.84 ^cC^	12.40 ^A^				
	PS	9.51 ^B^	3.83 ^bC^	12.86 ^aA^	12.32 ^A^				
BA	Control	ND	ND	ND	ND	-	-	-	-
	LP	ND	ND	ND	ND				
	PS	ND	ND	ND	ND				
NH_3_-N	Control	22.09 ^C^	5.60 ^D^	57.44 ^aA^	42.53 ^aB^	0.64	<0.001	<0.001	0.004
	LP	21.80 ^C^	3.53 ^D^	41.17 ^bA^	31.40 ^bB^				
	PS	19.52 ^C^	5.08 ^C^	56.44 ^aA^	41.14 ^aB^				
V-score	Control	91.31	92.71 ^b^	89.51 ^b^	90.00	0.22	<0.001	0.019	0.001
	LP	91.50 ^C^	95.21 ^abA^	93.09 ^aB^	90.00 ^D^				
	PS	91.58 ^B^	97.76 ^aA^	88.71 ^bC^	90.00 ^BC^				

Means within the same row (A–D) or within the same column (a–c) with different superscripts differ significantly from each other (*p* < 0.05). SEM, standard error of the mean; ND, not detected. ^1^ LA, lactic acid (g kg^−1^ DM); AA, acetic acid (g kg^−1^ DM); PA, propionic acid (g kg^−1^ DM); BA, butyric acid (g kg^−1^ DM); NH_3_-N, ammonia nitrogen (g kg^−1^ TN); V-score, score used to evaluate the silage quality according to the volatile fatty acid and NH_3_-N contents; TN, total nitrogen; DM, dry matter. ^2^ LP, *Lactobacillus plantarum* inoculant; PS, potassium sorbate. ^3^ D, effect of ensiling days; T, effect of treatment; D × T, interaction between ensiling days and treatment.

**Table 4:** Chemical compositions and in vitro digestibility of the total mixed ration silages after 30 days of ensiling.

Item ^1^	Treatment ^2^			SEM	*p*-Value
	Control	LP	PS		
Chemical compositions					
Dry matter (g kg^−1^ FM)	487.57	480.56	481.02	3.54	0.732
Crude protein (g kg^−1^ DM)	150.40	154.20	153.60	1.06	0.331
Water soluble carbohydrate (g kg^−1^ DM)	9.03 ^B^	7.03 ^B^	29.01 ^A^	3.59	<0.001
Neutral detergent fiber (g/kg (g kg^−1^ DM)	366.29	347.68	342.78	8.40	0.548
Acid detergent fiber (g kg^−1^ DM)	231.43	222.87	218.45	6.91	0.789
In vitro degradability					
DMD (g kg^−1^)	579.90	606.71	608.87	6.37	0.113
NDFD (g kg^−1^)	355.29	354.55	330.79	21.84	0.902
In vitro gas production parameters					
V_24h_ (mL)	57.15	57.01	56.57	0.72	0.957
V_48h_ (mL)	65.68	65.10	65.04	0.80	0.951
V(∞) (mL)	64.10	63.74	63.43	0.79	0.955
*k* (mL·h^−1^)	3.60	3.69	3.58	0.08	0.861

Means within the same row (A,B) with different superscripts differ significantly from each other (*p* < 0.05). SEM, standard error of the mean. ^1^ FM, fresh matter; DM, dry matter; DMD, dry matter digestibility; NDFD, neutral detergent fiber digestibility; V_24h_, 24-h cumulative gas production; V_48h,_ 48-h cumulative gas production; V(∞), maximal cumulative gas production; *k*, maximum gas production rate. ^2^ LP, *Lactobacillus plantarum* inoculant; PS, potassium sorbate.

**Table 5:** Changes in fermentative characteristics and chemical compositions of TMR silages after exposure to air.

Item ^1^	Treatment ^2^	Days of Air Exposure					SEM	*p*-Value ^3^		
		0	1	3	5	7		D	T	D × T
Fermentative characteristics										
pH	Control	4.26 ^bC^	4.28 ^bC^	4.28 ^bC^	4.53 ^B^	7.52 ^aA^	0.01	<0.001	<0.001	<0.001
	LP	4.25 ^bC^	4.28 ^bC^	4.31 ^bC^	4.56 ^B^	7.51 ^aA^				
	PS	4.59 ^a^	4.63 ^a^	4.65 ^a^	4.68	4.70 ^b^				
LA	Control	75.44 ^aA^	71.51 ^A^	71.55 ^abA^	71.55 ^A^	42.46 ^B^	1.17	0.006	0.001	0.178
	LP	69.39 ^aAB^	67.69 ^AB^	80.73 ^aA^	72.89 ^A^	48.67 ^B^				
	PS	56.23 ^b^	56.16	44.22 ^b^	51.16	50.84				
AA	Control	12.06 ^A^	11.20 ^A^	9.41 ^A^	4.80 ^bB^	2.61 ^B^	0.35	<0.001	0.172	0.224
	LP	12.25 ^A^	10.93 ^AB^	11.46 ^A^	5.64 ^abC^	6.02 ^BC^				
	PS	12.39 ^A^	11.45 ^AB^	7.85 ^C^	8.73 ^aBC^	7.47 ^C^				
PA	Control	12.51	11.91	10.82	10.75	9.48 ^b^	0.32	0.224	0.178	0.907
	LP	12.41	11.20	12.63	12.30	11.19 ^a^				
	PS	12.32	11.37	9.47	9.74	9.28 ^b^				
BA	Control	ND	ND	ND	ND	ND	-	-	-	-
	LP	ND	ND	ND	ND	ND				
	PS	ND	ND	ND	ND	ND				
NH_3_-N	Control	42.53 ^aB^	47.69 ^abB^	41.71 ^B^	44.30 ^B^	135.85 ^aA^	2.20	<0.001	0.001	<0.001
	LP	31.40 ^bB^	39.74 ^bB^	32.88 ^B^	53.18 ^B^	164.96 ^aA^				
	PS	41.14 ^a^	52.15 ^a^	44.24	41.51	44.04 ^b^				
V-score	Control	90.00 ^A^	89.84 ^A^	90.65 ^A^	89.89 ^A^	67.90 ^abB^	0.75	<0.001	0.002	<0.001
	LP	90.00 ^A^	90.00 ^A^	90.00 ^A^	89.37 ^A^	54.97 ^bB^				
	PS	90.00	89.45	90.80	90.05	89.60 ^a^				
Chemical compositions										
DM	Control	487.57	492.51	494.73	500.26	498.86	2.33	0.466	0.058	0.918
	LP	480.56	483.04	489.44	482.92	476.77				
	PS	481.02	489.89	499.81	499.66	504.83				
CP	Control	150.40 ^A^	150.88 ^A^	152.19 ^bA^	153.71 ^A^	144.00 ^bB^	0.39	0.003	0.015	<0.001
	LP	154.20 ^AB^	151.60 ^B^	157.18 ^aA^	155.84 ^AB^	146.07 ^bC^				
	PS	153.60 ^AB^	154.03 ^AB^	149.20 ^bC^	151.04 ^BC^	155.46 ^aA^				
WSC	Control	9.03 ^b^	11.71 ^b^	8.76 ^b^	7.92 ^b^	8.78 ^c^	0.35	0.037	<0.001	0.202
	LP	7.03 ^bC^	8.11 ^bBC^	6.91 ^bC^	10.22 ^bAB^	11.83 ^bA^				
	PS	29.01 ^a^	32.86 ^a^	27.18 ^a^	29.34 ^a^	30.20 ^a^				

Means within the same row (A–C) or within the same column (a–c) with different superscripts differ significantly from each other (*p* < 0.05). SEM, standard error of the mean. ^1^ LA, lactic acid (g kg^−1^ DM); AA, acetic acid (g kg^−1^ DM); PA, propionic acid (g kg^−1^ DM); BA, butyric acid (g kg^−1^ DM); NH_3_-N, ammonia nitrogen (g kg^−1^ TN); V-score, score used to evaluate the silage quality according to the volatile fatty acid and NH_3_-N contents; TN, total nitrogen; FM, fresh matter; DM, dry matter (g kg^−1^ FM); CP, crude protein (g kg^−1^ DM); WSC, water-soluble carbohydrate (g kg^−1^ DM). ^2^ LP, *Lactobacillus plantarum* inoculant; PS, potassium sorbate. ^3^ D, effect of aerobic exposure day; T, effect of treatment; D × T, interaction between aerobic exposure day and treatment.
